# Dose-Dependent Rescue of KO Amelogenin Enamel by Transgenes *in Vivo*

**DOI:** 10.3389/fphys.2017.00932

**Published:** 2017-11-16

**Authors:** Felicitas B. Bidlack, Yan Xia, Megan K. Pugach

**Affiliations:** ^1^Forsyth Institute, Cambridge, MA, United States; ^2^Department of Developmental Biology, Harvard School of Dental Medicine, Boston, MA, United States

**Keywords:** enamel development, transgenic, knockout, amelogenin, mineralization

## Abstract

Mice lacking amelogenin (KO) have hypoplastic enamel. Overexpression of the most abundant amelogenin splice variant M180 and LRAP transgenes can substantially improve KO enamel, but only ~40% of the incisor thickness is recovered and the prisms are not as tightly woven as in WT enamel. This implies that the compositional complexity of the enamel matrix is required for different aspects of enamel formation, such as organizational structure and thickness. The question arises, therefore, how important the ratio of different matrix components, and in particular amelogenin splice products, is in enamel formation. Can optimal expression levels of amelogenin transgenes representing both the most abundant splice variants and cleavage product at protein levels similar to that of WT improve the enamel phenotype of KO mice? Addressing this question, our objective was here to understand dosage effects of amelogenin transgenes (*Tg*) representing the major splice variants M180 and LRAP and cleavage product CTRNC on enamel properties. Amelogenin KO mice were mated with M180*Tg*, CTRNC*Tg* and LRAP*Tg* mice to generate M180*Tg* and CTRNC*Tg* double transgene and M180*Tg*, CTRNC*Tg*, LRAP*Tg* triple transgene mice with transgene hemizygosity (on one allelle) or homozygosity (on both alleles). Transgene homo- vs. hemizygosity was determined by qPCR and relative transgene expression confirmed by Western blot. Enamel volume and mineral density were analyzed by microCT, thickness and structure by SEM, and mechanical properties by Vickers microhardness testing. There were no differences in incisor enamel thickness between amelogenin KO mice with three or two different transgenes, but mice homozygous for a given transgene had significantly thinner enamel than mice hemizygous for the transgene (*p* < 0.05). The presence of the LRAP*Tg* did not improve the phenotype of M180*Tg*/CTRNC*Tg*/KO enamel. In the absence of endogenous amelogenin, the addition of amelogenin transgenes representing the most abundant splice variants and cleavage product can rescue abnormal enamel properties and structure, but only up to a maximum of ~80% that of molar and ~40% that of incisor wild-type enamel.

## Introduction

Tooth enamel forms through appositional growth in an organic matrix that is secreted in daily increments until the full thickness of the crown is reached. During this first, secretory, stage of enamel formation, enamel crystallites grow primarily in length. Once the final enamel thickness is attained, the mineral content increases as crystallites grow in thickness, while the organic phase is removed in a highly controlled way. The enamel matrix facilitates mineralization and organization, and is transient in abundance as well as composition. Starting out with a ratio of 70 wt% organic matter and water, and 30 wt% mineral (Smith, 1998) the ratio of organic matrix to mineral changes over the course of enamel development and reaches 95% mineral content, with about 1–2% organic matter retained in completed enamel. During the secretory stage, the full-length enamel matrix molecules amelogenin, ameloblastin, and enamelin are cleaved upon their secretion by matrix metalloproteinase 20 (MMP20). After the secretory stage, a second enzyme, kallikrein 4 (KLK-4) is active to further cleave the matrix proteins and allow for the removal of matrix until only a very small amount remains, which is important for the mechanical properties through the control of crack propagation. The three structural enamel matrix proteins and the alternative splice product of amelogenin, leucine rich amelogenin protein (LRAP) have been described (Smith, 1998; Bartlett, 2013; Tarasevich et al., 2015; Lacruz et al., 2017). Yet, is not resolved what the *in vivo* function of these matrix components is, what role the full-length molecules, their alternative splice products as well as their cleavage products play for the control of mineral phase, crystallite shape and orientation, pH regulation, and potentially feed-back to ameloblasts (Lacruz et al., 2017).

The organic enamel matrix comprises the three structural matrix proteins amelogenin, enamelin, and ameloblastin, with amelogenin accounting for 90 wt% of the composition. However, the amelogenin primary RNA transcript is extensively alternatively spliced to produce 16 amelogenin isoforms reported (Bartlett et al., 2006). It is not clear, whether these isoforms are critical for enamel formation, or what their roles are in amelogenesis. The most abundant of these isoforms are the full-length molecule of 180 amino acids and the 59-amino acid long LRAP, leucine-rich amelogenin protein, which consist of the 33 N-terminal and 26 C-terminal amino acids, but lacks the hydrophobic core of the full-length molecule.

Ameloblasts secrete the full-length 180 amino acid sized amelogenin, which shortly thereafter is cleaved by the metalloproteinase MMP20, beginning from the C-terminus. The most abundant cleavage product is 167 amino acids long, and referred here to as CTRNC. All three structural matrix proteins are required for proper enamel formation and the AmelxKO enamel is hypoplastic, with no prismatic architecture. In order to determine the roles of the most abundant amelogenin isoforms, transgenic mice have been developed that overexpress (a) the full-length amelogenin M180, (b) the major amelogenin cleavage product CTRNC, and (c) LRAP in both C57BL6/J wild-type and *Amelx*KO genetic backgrounds (see Table 1 for abbreviations).

**Table 1:** Abbreviations for amelogenin protein, transgenes, and mouse models.

M180—M180*Tg*—M180*Tg*/KO	Full-length amelogenin isoform of 180 amino acids—M180 transgene—mouse model with M180 transgene in amelogenin null background
CTRNC—CTRNC*Tg*—CTRNC*Tg*/KO	Cleaved (M180) amelogenin of 167 amino acids—CTRNC transgene—mouse model with CTRNC transgene in amelogenin null background
LRAP—LRAP*Tg*—LRAP*Tg*/KO	Amelogenin isoform of 59 amino acids—LRAP transgene—mouse model with LRAP transgene in amelogenin null background

Addition of LRAP*Tg* to M180*Tg* in the amelogenin KO model improves the thickness and structure of enamel, suggesting that transgenes have a complementary function (Gibson et al., 2011). Among amelogenin isoforms, M180 by itself is sufficient for the formation of normal mechanical properties and prism patterns in enamel. Yet, additional amelogenin splice products are required to restore enamel thickness (Gibson et al., 2011; Snead et al., 2011; Pugach et al., 2013). The overexpression of CTRNC*Tg* and LRAP*Tg* together improved significantly the enamel phenotype of LRAP*Tg*/KO and CTRNC*Tg*/KO mouse enamel, however enamel microhardness was recovered only when M180*Tg* was expressed, alone or with LRAP*Tg*. Expression of LRAP and CTRNC together provides all three regions of the amelogenin protein N-terminus, C-terminus and hydrophobic core further improved the phenotype to reach normal WT enamel thickness and prism organization in the reported mouse model (Chen et al., 2003; Pugach et al., 2010; Xia et al., 2016). Enamel phenotypes in M180*Tg*/KO, CTRNC*Tg*/KO, LRAP*Tg*/KO, and double transgenic mice have been heterogeneous. This is due to varying transgene dosages and suggests a cumulative effect on improving the *Amelx*KO enamel phenotype (Xia et al., 2016).

The importance of the ratio between cleaved and uncleaved amelogenin has been previously reported (Shin et al., 2014). In order to determine the optimal ratio of amelogenin major cleavage products and splice variants required for normal enamel structure, thickness, and mechanical properties, we generated double and triple transgenic mice with two different transgene dosages in KO backgrounds.

## Materials and methods

### Generation of transgenic mice and genotyping

To generate M180*Tg*/CTRNC*Tg*/LRAP*Tg*/KO and M180*Tg*/CTRNC*Tg*/KO mice, animals overexpressing M180, CTRNC, and LRAP transgenes from the bovine amelogenin promoter were mated with male Amelx–/0 mice. After two generations of mating, both male and female mice were genotyped using PCR primers to detect transgenes as well as amelogenin WT and Amelx−/− (KO) DNA (Gibson et al., 2001, 2007; Chen et al., 2003; Pugach et al., 2010). Four different genotypes were generated with either 2 (M180*Tg* and CTRNC*Tg*) or 3 (M180*Tg*, CTRNC*Tg*, and LRAP*Tg*) transgenes, and either homozygous (++) or hemizygous (+/−) for the transgenes (*N* = 3–5 mice per genotype). PCR primers for M180*Tg*, CTRNC*Tg*, LRAP*Tg*, and KO mice have been previously published (Gibson et al., 2001; Chen et al., 2003; Li et al., 2008; Pugach et al., 2010). Relative copy number determined transgene homo- (++ = on both alleles) vs. hemizygosity (+ = on one allelle), and was determined by qPCR analysis using M180, CTRNC, and LRAP *Tg*-specific probes designed by Transnetyx (Cordova, TN) (Table 2). WT, KO, and hemizygous (+/−) M180*Tg*/LRAP*Tg*/KO mice, which have been previously reported were used as controls (Gibson et al., 2001, 2011).

**Table 2:** Mouse genotypes (KO and transgenic status) as determined by PCR for genotyping for endogenous amelogenin and qPCR for measuring relative copy number of the three transgenes, using transgene-specific probes against M180*Tg*, CTRNC*Tg*, and LRAP*Tg*.

**Genotypes**	***Amelx*KO**	**M180*Tg* (180aa)**	**CTRNC*Tg* (167aa, no C-terminus)**	**LRAP*Tg* (59aa, C- and N-only)**
1: M180*Tg*/CTRNC*Tg*/LRAP*Tg*/KO++	−/o or −/−	++	++	++
2: M180*Tg*/CTRNC*Tg*/LRAP*Tg*/KO+	−/o or −/−	+	+	+
3**:** M180*Tg*/CTRNC*Tg*/KO++	−/o or −/−	++	++	–
4: M180*Tg*/CTRNC*Tg*/KO+	−/o or −/−	+	+	–
Control: M180*Tg*/LRAP*Tg*/KO+	−/o or −/−	+	–	+
Control: *Amelx*KO	−/o or −/−	–	–	–
Control: WT	+/o or +/+	–	–	–

### Western blot analysis

**Figure 1:**
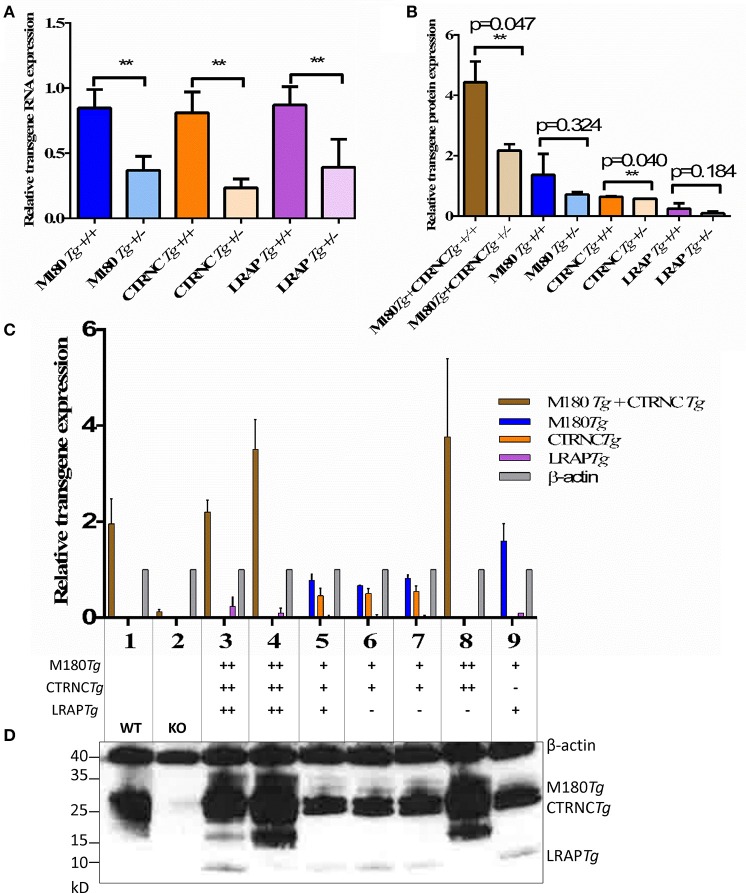
Relative amelogenin transgene DNA and protein expression in double and triple transgenic/*Amelx*KO mice. **(A)** Relative transgene copy number of homozygous vs. hemizygous M180*Tg*, CTRNC*Tg*, and LRAP*Tg* as determined by qPCR analyses with transgene-specific probes designed and tested by Transnetyx (Cordova, TN) (^**^indicates significant difference, *p* < 0.0001). **(B–D)** Western blot of *Amelx* protein expression using anti-Amelogenin FL-191 (Santa Cruz), with β-actin control (Sigma) in transgenic 5 day-old developing molars. **(B)** Relative transgene protein expression of homozygous vs. hemizygous M180*Tg*, CTRNC*Tg*, and LRAP*Tg* as determined by Western blot analyses as measured by ImageJ using β-actin to normalize. Since we could not differentiate between M180*Tg* and CTRNC*Tg* in lanes 1, 3, 4, and 8, M180*Tg* and CTRNC*Tg* expression intensities were pooled when quantifying. Due to the lack of Mmp20 in lanes 5, 6, and 7, we were able to differentiate between M180Tg and CTRNC*Tg*. (*n* = 3 blots, ^**^indicates significant difference, *p* < 0.005). **(C)** Relative transgene protein expression for different genotypes as determined by Western blot analyses as measured by ImageJ using β-actin to normalize. Lane 1, WT; Lane 2, *Amelx*KO; Lane 3, M180*Tg*/CTRNC*Tg*/LRAP*Tg*/KO++; Lane 4, M180*Tg*/CTRNC*Tg*/KO++; Lane 5, M180*Tg*/CTRNC*Tg*/KO+ (*Mmp20*KO background control); Lane 6, M180*Tg*/CTRNC*Tg*/LRAP*Tg*/KO++ (*Mmp20*KO background control); Lane 7, M180*Tg*/CTRNC*Tg*/LRAP*Tg*/KO+ (*Mmp20*KO background control); Lane 8, M180*Tg*/CTRNC*Tg*/KO++; Lane 9, M180*Tg*/LRAP*Tg*/KO+. **(D)** Western blot with primary antibodies anti-Amelogenin and anti-β-actin, with lane numbers corresponding to genotypes in **(C)**. In lane 1, molars had a smear of amelogenin protein between 17 and 20 kD representing most of the WT splice variants and cleavage products expressed during the secretory stage, which are absent in the *Amelx*KO lane 2. The M180*Tg* band is visible at ~25 kD in all lanes except lane 2. The CTRNC*Tg* is visible ~22 kD in lanes 5, 6, and 7, since the absence of *Mmp20* prevented its proteolytic degradation. The LRAP*Tg* band is visible ~7 kD in lanes 3, 6 7, and 9, but below the detection level in lane 1. The β-actin loading control bands is visible in all lanes ~40 kD.

To analyze expression of endogenous and transgenic amelogenin in M180*Tg*/CTRNC*Tg*/LRAP*Tg*/KO and M180*Tg*/CTRNC*Tg*/KO male and female mice and controls, first molars were harvested from 5-day-old mouse pups (with ameloblasts in the secretory stage) and protein was extracted. Equal amounts of protein were loaded in each lane and run on a 4–20% SDS-PAGE gel (BioRad). Membranes were immunoblotted with an antibody against full-length *Amelx* (FL-191, Santa Cruz Biotechnology, Santa Cruz, CA) and against β-actin (A2103, Sigma-Aldrich, St. Louis, MO), with a goat anti-rabbit secondary antibody (Santa Cruz Biotechnology). Relative transgene expression was confirmed by Western blot, and mice with the same genotypes but *Mmp20*KO background were used as controls (Figure 1). β-actin was used as the loading control to quantify relative transgene expression levels.

### Tooth sample preparation and analyses of enamel

Six 6-week-old male and female adult hemi-mandibles were dissected and fixed in Zinc-formalin for 24 h, then rinsed and transferred to 50% ethanol. Samples were first analyzed by μCT, then dehydrated in a graded ethanol series and embedded in LR-White (Electron Microscopy Sciences, Hatfield, PA, USA) for micro-hardness testing, and lastly SEM analyses.

#### Enamel mineral density

Enamel mineral density was determined by μCT and compared between standardized regions of interest in incisor and molar enamel in mutant and control mice as described previously (Pugach et al., 2013). Hemimandibles with soft tissues removed were scanned in a μCT-40 (Scanco, Brüttisellen, Switzerland) at 70 kV, 114 mA, and 6 μm resolution. Images were processed with μCT-40 evaluation software and FIJI (https://fiji.sc/) was used to orient the mandibles in a standardized way based on anatomical landmarks to clearly observe and compare enamel mineralization in two locations: (1) In the first molar, in a coronal plane through the distal root and, extending this plane, in the early maturation stage of the developing incisor and, (2) in the maturation stage incisor, in coronal plane through the mandible proximal to the point of tooth eruption, that is where the incisor is still completely enclosed by bone.

#### Enamel hardness

Enamel hardness was determined from first molars on LR White embedded samples that were polished on M3 polishing film (Precision Surfaces International, Houston Texas, USA) to 0.3 μm grit size in parasagittal plane. The polished samples were tested for enamel microhardness on an M400 HI testing machine (Leco, St. Joseph, MI) with a load of 10 g for 5 s with a Vickers tip, applying 20 indentations per sample on at least four teeth per group, with data averaged per group.

#### Enamel thickness analyses

Enamel thickness analyses were performed on samples subsequently to micro-hardness testing. The sample surface was etched with 0.1 M phosphoric acid for 15 s, gold coated, and imaged with a Zeiss Evo LS 10 SEM at 15 kV, 6–8 mm WD, and 120 pA probe current. Enamel thickness was measured in first molars and incisors on images taken at 1000X at the mesial side of the first molar using lateral enamel and in incisors in the area underneath the mesial first molar root tip, respectively, in at least six samples per genotype.

#### Enamel microstructure

Enamel microstructure was analyzed on both para-sagittal sections prepared for enamel thickness measurements as well as coronal sections through the mesial root of the first molar and visualized at 2000X and higher magnification.

#### Toluidine blue staining

Toluidine Blue staining was applied to visualize organic matter in enamel. The same samples analyzed for microstructure by SEM were used for toluidine blue staining. The gold coating was polished off, the sample mounted on a glass slide, and a thin section prepared through polishing to a final thickness of ~100 μm. A 1% toluidine blue solution was used for 3 min, rinsed off and samples air-dried before viewing in a Leica upright microscope.

### Statistical methods

We used ANOVA with the Tukey *post-hoc* test to detect differences (*p* < 0.05) in RNA expression, protein expression, enamel thickness, and microhardness between groups of teeth analyzed for enamel thickness and Vickers microhardness (GraphPad Software, San Diego, CA, USA).

## Results

### Relative amelogenin transgene protein expression

qPCR and Western blot analyses confirmed that we generated four different genotypes with either two transgenes (M180 & CTRNC) or three transgenes (M180, CTRNC, LRAP) and homozygous (++) or hemizygous (+) expression in KO mice (Table 2). As expected, qPCR data confirm that the transgene copy numbers are higher in the homozygous than in hemizygous transgenic mice. However, the double transgene expression level of M180 with only CTRNC, without LRAP, is decreased in the homozygous transgenic compared to the triple homozygous transgenic containing LRAP (Figure 1A). Western Blot analyses show that in developing day-5 molars, that in the triple transgene on *Amelx*KO background most of the splice variants and cleavage products are expressed during the secretory stage and visible as bands between 17 and 20 kD for M180 and CTRNC, and the LRAP band around 7 kD (Figure 1B). The LRAP*Tg* expression in triple transgenic/*Amelx*−/− mice is higher in the homozygous mouse compared to hemizygous, as expected, but also higher than that in WT molars.

### Relative enamel mineral density

**Figure 2:**
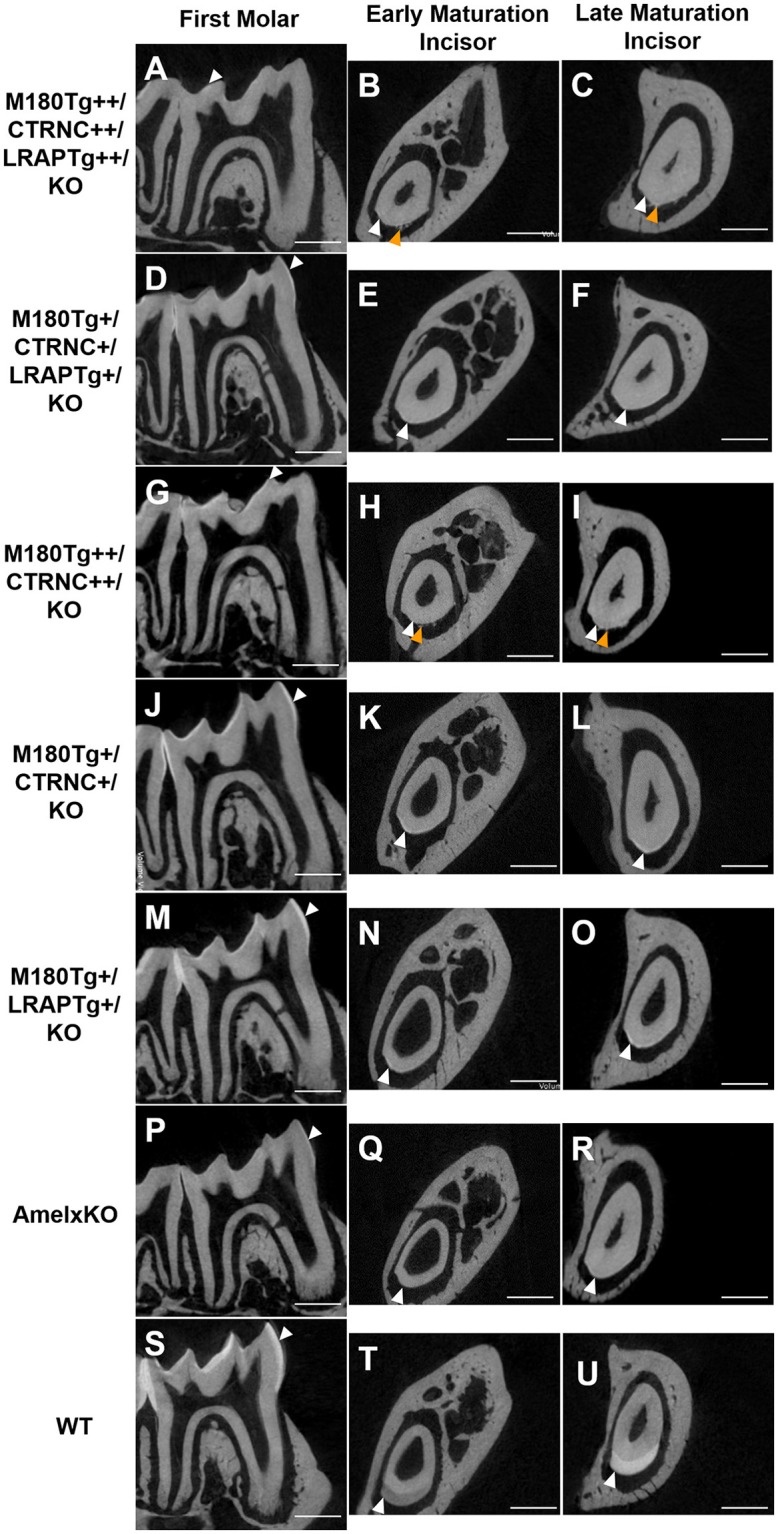
MicroCT analyses of adult molar and incisor enamel. First column: first molars in mid sagittal plane. Middle column: Incisor enamel in coronal plane through the distal root of the first molar, representing early maturation stage. Last column: Incisor enamel in coronal plane adjacent and mesial to the first molar, representing maturation stage enamel. White arrowheads point to mineralized enamel layers visible in molars and incisors. WT seen in last row **(S–U)**; **(P–R)** *Amelx*KO with little enamel on molars and no enamel in the incisor enamel. **(A–C)** homozygous triple transgene M180*Tg*/CTRNC*Tg*/LRAP*Tg*/KO++, showing the thinnest enamel layer compared to all samples shown on both molars and incisors. **(D–F)** Hemizygous triple transgene M180*Tg*/CTRNC*Tg*/LRAP*Tg*/KO+ and **(M–O)** hemizygous double transgene M180*Tg*/LRAP*Tg*/KO+ with thicker enamel than all other transgene phenotypes shown, but thinner than WT. **(G–I)** double transgene M180*Tg*/CTRNC*Tg*/KO++ with a thin layer of enamel as is also seen in **(J–L)** M180*Tg*/CTRNC*Tg*/KO+. Ectopic depositions (orange arrowheads) were visible in incisors of homozygous transgenes M180*Tg*/CTRNC*Tg*/LRAP*Tg*/KO++ and M180*Tg*/CTRNC*Tg*/KO++ **(A–C, G–I)**. Scale bars, 500 μm.

Results from μCT analyses of incisor and molar enamel show differences between the five different genotypes and compared to WT and *Amelx*KO (Figure 2). In no transgene combination was the enamel thickness of WT enamel achieved. In addition, mineral density was decreased in both molar and incisor enamel with homozygous (++) transgene expression (Figures 2A–C, G–H), compared to hemizygous (+) transgene expression (Figures 2D–F, J–O). The enamel layer seen in the homozygous triple transgene M180*Tg*/CTRNC*Tg*/LRAP*Tg*/KO++ (Figures 2A–C) is also thinner than in the hemizygous double transgene M180*Tg*/CTRNC*Tg*/KO+ (Figures 2J–L) and M180*Tg*/LRAP*Tg*/KO+ (Figures 2M–O) on both molar and incisor. We observed ectopic depositions in mice with excess CTRNC*Tg* (++) without LRAP*Tg* (Figures 2A–C, G–I).

### Enamel thickness analyses

Based on SEM data (Table 3), incisor enamel of triple transgenic homozygous (++) mice was statistically (*p* < 0.05) thicker than triple transgenic enamel with less transgene expression (+), indicating a dosage effect. This effect is not seen in the molars. However, a difference between double and triple homozygous transgenic mice was clear in molar enamel, which was thicker in the triple transgene (*p* = 0.038), relating molar enamel thickness to the presence of LRAP in the matrix (Table 3). Interestingly, enamel thickness is highest in hemizygous double transgenic mice, which is when the matrix lacks LRAP but does contain M180 and CTRNC.

**Table 3:** Enamel thickness and hardness of adult mandibular molars and incisors.

**Genotype**	***N***	**Incisor enamel thickness (μm)**	**Molar enamel thickness (μm)**	**Molar enamel hardness (GPa)**
Wild-type (C57BL/6J)	12	119.7 (4.3)^x^	61.6 (3.8)^x^	257.4 (64.6)
Amelogenin KO	12	19.1 (5.2)^w^	11.5 (1.2)^w^	217.2 (60.0)
M180*Tg*/CTRNC*Tg*/LRAP*Tg*/KO (++)	4	33.7 (10.9)^w x^	33.4 (17.2)^w x^	238 (87.4)
M180*Tg*/CTRNC*Tg*/LRAP*Tg*/KO (+)	4	28.1 (4.5)^w^	33.4 (10.9)^w x^	200.4 (61.6)
M180*Tg*/CTRNC*Tg*/KO (++)	4	33.7 (11.4)^w x^	23.7 (4.1)^w x 3o^	150 (52.3)
M180*Tg*/CTRNC*Tg*/KO (+)	4	41.6 (10.1)^w x 3t^	36.0 (7.2)^w x 2o^	294 (108.6)^x^
M180*Tg*/LRAP*Tg*/KO (+)^a^	6	45.3 (4.5)^w x 3o 3t 2o^	51.4 (7.6)^w x 3o 3t 2o 2t^	265.0 (59.9)
CTRNC*Tg*/LRAP*Tg*/KO (+)^b^	6	53.1 (14.7)^w x 3o 3t 2o 2t^	41.4 (13.0)^w x 3o 3t 2o^	190.2 (65.3)^w 2t^
M180*Tg*/KO (+)^c^^,^^d^	6	26.8 (9.6)^w 2t^	43.0 (7.5)^w x 3o 3t 2o^	291.2 (39.8)^x 3t^
CTRNC*Tg*/KO (+)^e^	6	28.1 (11.7)^w x 2t^	19.6 (3.9)^w x 3o 3t 2t^	208.0 (75.0)^w 2t^
LRAP*Tg*/KO (+)^f^^,^^g^	6	24.1 (7.3)^w3o 2o 2t^	19.0 (2.2)^w x 3o 3t 2t^	193.3 (66.5)^2t^

*Significant differences (p < 0.05) are indicated as follows: ^w^from WT; ^x^from Amelogenin KO; ^3o^from M180Tg/CTRNCTg/LRAPTg/KO (++); ^3t^from M180Tg/CTRNCTg/LRAPTg/KO (+); ^2o^from M180Tg/CTRNCTg/KO (++); ^2t^from M180Tg/CTRNCTg/KO (+)*.

a Gibson et al. (2011);

b *Xia et al. (2016)*,

c *Li et al. (2008)*,

d *Pugach et al. (2013)*,

e *Pugach et al. (2010)*,

f *Chen et al. (2003)*,

g *Gibson et al. (2009)*.

### Enamel microhardness

Vickers microhardness data of the four different genotypes are shown in Table 3. The enamel phenotypes are highly variable due to a mosaic appearance of properties including ectopic depositions and resulted in such high standard deviations of microhardness data that differences in hardness were not statistically significant between groups. However, molar double transgenic hemizygous enamel was harder than homozygous double transgenic molar enamel (*p* < 0.05).

### Enamel structure analyses by SEM and organic matter content

**Figure 3:**
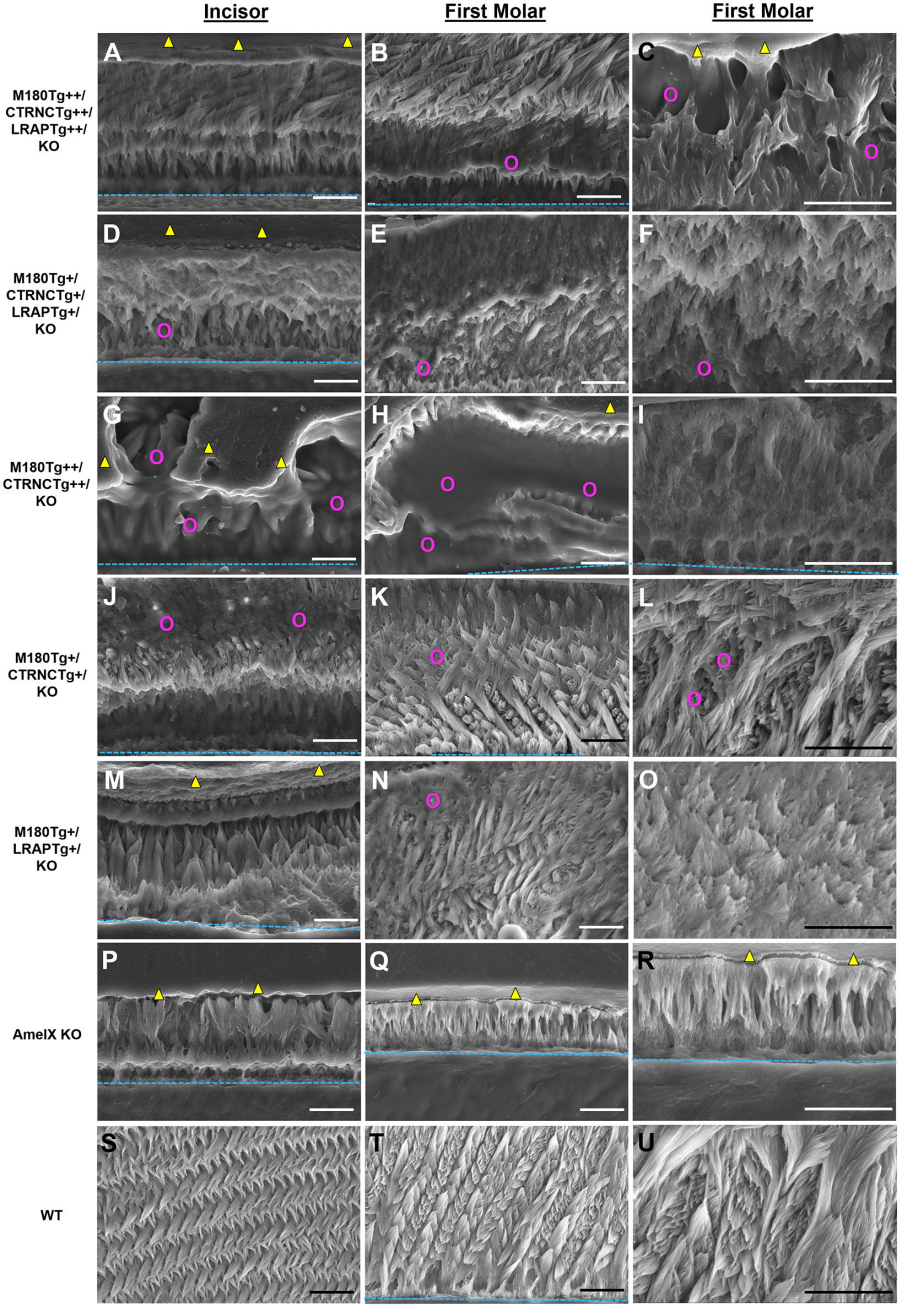
SEM analysis of double and triple transgenic/KO incisor and molar enamel. Polished and etched sections through mandibular incisor enamel and first molars imaged by scanning electron microscopy with secondary electron detection. **(A–C)** Homozygous triple transgenic M180*Tg*/CTRNC*Tg*/LRAP*Tg*/KO++, **(D–F)** hemizygous triple transgenic M180*Tg*/CTRNC*Tg*/LRAP*Tg*/KO+, **(G–I)** homozygous double transgenic M180*Tg*/CTRNC*Tg*/KO++, **(J–L)** hemizygous double transgenic M180*Tg*/CTRNC*Tg*/KO+ enamel, and **(M–O)** hemizygous double transgenic M180*Tg*/LRAP*Tg*/KO+, **(P–R)** AmelX null, **(S–U)** WT enamel. First column: Incisors, scale bars 10 μm. Second column: Molars, scale bars 10 μm. Third column: Molars, scale bars 10 μm. Yellow triangles: LR White resin; turquoise dashed lines: DEJ; magenta colored circles: organic matrix in forming enamel.

**Figure 4:**
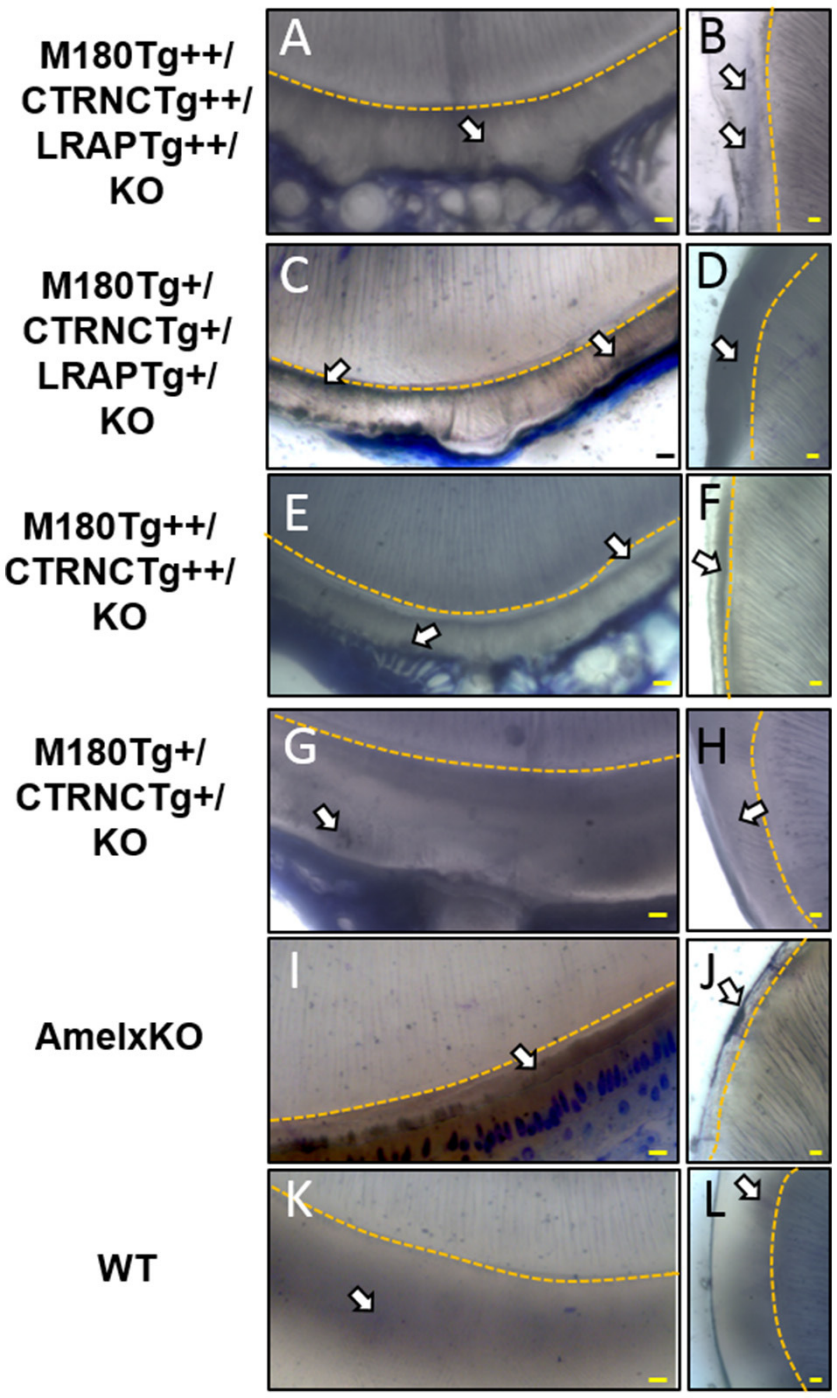
Toluidine Blue staining of LR White embedded samples processed further after SEM analyses. **(A,B)** Homozygous triple transgenic enamel M180Tg/CTRNCTg/LRAPTg/KO++, **(C,D)** hemizygous triple transgenic M180Tg/CTRNCTg/LRAPTg/KO+, **(E,F)** homozygous double transgenic M180Tg/CTRNCTg/KO++, **(G,H)** hemizygous double transgenic M180Tg/CTRNCTg/KO+ enamel, **(I,J)** AmelX null, **(K,L)** WT enamel. Coronal sections through incisors (first column) and molars (second column). Nuclei and organic matter stained blue and marked by arrows, DEJ highlighted in yellow. Scale bars, 10 μm. Magnification 400X.

The incisors of all transgene combinations analyzed here differ from WT enamel in both an excess of retained organic matter and a distinctly layered structural organization of inner and outer enamel. This layering is clearly seen in the homozygous triple transgene M180Tg/CTRNC*Tg*/LRAP*Tg*/KO ++ incisor and molar, with layers distinguished by the pattern and clarity of prism decussation and amount of organic matrix (Figures 3A–C, 4B). In comparison, the hemizygous triple transgene expression appeared to result in fewer layers in the incisor (Figure 3D) and less organic matrix retention in molars (Figures 3E,F), while maintaining prism organization (Figures 3D–F). Toluidine blue staining shows more homogeneity within the molar compared to the incisor where we see organic matter close to the DEJ and near the enamel surface (Figures 4C,D). In contrast, homozygous double transgenic enamel had extensive disruption of structural organization, appeared to contain more organic matrix and showed only rudimentary prismatic organization (Figures 3G–I, 4E,F). Ectopic depositions were observed in mice with homozygous expression of the CTRNC transgene (Figure 3G).

Retained organic matrix was observed in incisor and molar enamel from all four genotypes, especially those with homozygous transgene expression of CTRNC*Tg* (Figures 3B,C,G,H, 4A–H). The inclusion of the LRAP*Tg* did not improve the structure of M180Tg/CTRNC*Tg* enamel in homozygous nor hemizygous transgenes. Consistent with μCT and SEM derived thickness data, the enamel of hemizygous double transgenic mouse molars M180*Tg*/CTRNC*Tg*/KO+ and M180*Tg*/LRAP*Tg*/KO+ are most similar to WT (Figures 3K,L,N,O,T,U). In a given animal the incisor enamel contains more retained organic matrix in the inner enamel, compared to the molars and WT as seen in SEM (Figures 3J,M,S), and toluidine blue stained samples (Figures 4G,K).

## Discussion

It has been shown previously that enamel prism decussation and 83% of thickness is recovered in the double hemizygous transgenic mouse M180*Tg*/LRAP*Tg*/KO model (Gibson et al., 2011). To better understand the *in vivo* role of full-length amelogenin vs. cleavage and alternative splice products, we generated four genotypes that differ from each other in their relative abundance of M180, its most abundant cleavage product CTRNC that lacks the C-terminus, and the most abundant alternative splice product LRAP, which contains only the N- and C-terminus of the full-length molecule, but not the hydrophobic core region. However, in the present study, enamel thickness did not rescue by more than 58%, and prism organization was not fully achieved in incisors (Figure 3; Table 3).

In normal enamel development, M180 is cleaved shortly after secretion. Mutations in either the C-terminus or N-terminus compromise enamel formation. There are five reported mutations in the *AMELX* C-terminal region, and six mutations in the N-terminal region, leading to thin, discolored, and hypoplastic enamel (Lagerström et al., 1991; Lagerström-Fermer et al., 1995; Lench and Winter, 1995; Kindelan et al., 2000; Sekiguchi et al., 2001; Greene et al., 2002; Hart et al., 2002; Kim et al., 2004; Cho et al., 2014). Mutations in the N-terminus cause self-assembly defects *in vitro* (Buchko et al., 2013). Amelogenin lacking the N-terminus does not form nanospheres *in vitro*, and *in vivo*, the enamel is thin with short crystallites and irregular enamel prisms, indicating the key role of the N-terminus in amelogenin self-assembly and crystallite elongation (Zhu et al., 2006).

Both recent *in vitro* and *in vivo* evidence suggest that CTRNC plays an important role in enamel mineralization (Kwak et al., 2009, 2011; Martinez-Avila et al., 2011; Xia et al., 2016). *In vitro* studies have suggested a role of full-length amelogenin in the control of mineral phase, specifically the stabilization of amorphous mineral phases such as amorphous calcium phosphate (ACP), and alignment of forming crystallites (Kwak et al., 2011). With cleavage of the full-length molecule, one would expect the transformation of ACP to hydroxyapatite to proceed. *In vivo*, amelogenin transcription and MMP20 activity regulate the ratio of M180 to its cleavage product. The choreographed interplay between amelogenin secretion, MMP20 activity, and the resulting abundance and ratio of M180 to CTRNC can provide a mechanism to regulate mineralization rate.

Our data show that the dose of transgene expression has a major effect on enamel formation as is seen in the comparison between homozygous double transgene of M180*Tg*++/CTRNC*Tg*++/KO and hemizygous transgene of M180 and CTRNC. This hemizygous transgene best rescues the structural organization and prism decussation of enamel. However, the produced enamel is softer than in any other transgene model used in this study. In contrast, the homozygous transgene provides excess protein that disrupts the process of both mineral phase regulation and crystallite alignment, as seen in the small platelets compared to the elongated crystals (Figure 3I) in the hemizygous enamel. The excess organic matrix in the homozygous M180*Tg*++/CTRNC*Tg*++/KO transgene remains in the extracellular space, covers enamel prisms and seems to prevent proper decussation resulting in diminished hardness (Figures 3G,H; Table 3). In addition, matrix secretion is disrupted in the homozygous transgene, resulting in ectopic depositions (Figures 2H,I, 3G) and decreased enamel thickness. This finding highlights the importance of sheer abundance of matrix components at a given time, and in relation to MMP20 activity, and is paralleled in the results for the homozygous transgene M180*Tg*++/CTRNC*Tg*++/LRAP*Tg*++, where also ectopic depositions are observed, excess organic material covering prisms and diminished enamel thickness (Figures 2, 3). The rate of enamel apposition and crown extension is higher in incisors compared to molars, which could contribute to the observed differences in structural organization, matrix deposition, and matrix removal between molars and incisors (Smith and Warshawsky, 1977).

Interestingly, enamel organization and prism decussation, as well as hardness is better rescued than in the homozygous transgene without LRAP, M180Tg++/CTRNCTg++. The capacity of LRAP to regulate mineral phase *in vitro*, specifically stabilize ACP, has been shown by Le Norcy et al. (2011). In contrast to the homozygous transgene model, a comparison between the hemizygous transgenic mice M180*Tg*+/CTRNC*Tg*+/LRAP*Tg*+ and M180*Tg*+/CTRNC*Tg*+ shows that the structural organization is much better without LRAP than when it is expressed uniformly with M180 and CTRNC. Our data further suggest that LRAP*Tg* overexpressed with M180*Tg* and CTRNC*Tg* does not affect enamel thickness (Table 3). These findings underscore the importance of other factors besides absolute abundance of matrix molecules, namely, the timing of transgene expression and the relative abundance of cleavage product and alternative splice products. While LRAP uses the same promoter *in vivo* as M180, it is not known how LRAP expression varies between stages of enamel development. In addition, the pH buffering effect of matrix components is relevant for mineralization rate and produced mineral phase.

A possible role of the central core domain of amelogenin is the buffering of pH during enamel mineralization and it has been suggested previously that amelogenins may act as a buffer to neutralize protons generated during enamel crystal formation in the secretory stage (Smith, 1998; Smith et al., 2005). Although the role of the central hydrophobic core remains elusive, seven amelogenesis imperfecta-causing mutations in this region are published to date (Aldred et al., 1992; Lench et al., 1994; Lench and Winter, 1995; Collier et al., 1997; Hart et al., 2000, 2002; Ravassipour et al., 2000; Sekiguchi et al., 2001; Greene et al., 2002). In all the transgene models presented here, enamel thickness is decreased, indicating abridged matrix secretion compared to normal enamel development in the WT, where the ending of amelogenin secretion coincides with both the attainment of the full enamel thickness and the enamel matrix becoming acidic (Smith, 1998). Amelogenins including the central hydrophobic core contain 14 histidine residues, which can bind protons such that a single amelogenin molecule can bind up to 15 protons *in vitro* (Ryu et al., 1998). Further support for a suggested role of amelogenin in pH regulation comes from data comparing ion channel expression in ameloblasts during secretory and maturation stage between WT and *Amelx*KO mice. In WT, secretory ameloblasts do not express the anion exchanger Ae2 basolaterally (Lyaruu et al., 2008; Bronckers et al., 2009). However, the expression of Ae2 in secretory ameloblasts of amelogenin KO mice indicate a mechanism to compensate for the lack of buffering in the absence of amelogenin through upregulation of Ae2 expression to secrete bicarbonate (Guo et al., 2015).

Taken together, our data suggest that all three domains of amelogenin play key roles in enamel formation and that the relative abundance over time is critical. The N- and C-termini of amelogenin, which are present in both the most abundant amelogenin (M180) and LRAP, are highly conserved and believed to have different but critical roles in enamel formation (Delgado et al., 2007). Our results support the hypothesis that the core domain affects enamel formation, in particular the aspects of enamel thickness *in vivo* through crystal elongation. This study highlights the need to appreciate the relative abundance of enamel matrix molecules and their role for pH regulation as a key factor of enamel formation. In conclusion, we have shown that excess amelogenin transgenes disrupted the process of enamel formation, likely through the disproportionate presence of amelogenin splice products and disruption of matrix removal. The presence of excess retained organic matrix, layering within enamel, and ectopic depositions in the mouse models studied, suggest that an optimal ratio between M180, CTRNC, and LRAP is critical for normal enamel structure, thickness, and hardness.

## Ethics statement

This study was carried out in accordance with the recommendations of Institute, Federal and Institutional Animal Care and Use Committee (IACUC) guidelines. The protocol was approved by the IACUC at Forsyth.

## Author contributions

MP and FB wrote the manuscript with contributions from all authors. MP, FB, and YX contributed to the design of the experiments. MP, FB, and YX performed and analyzed experiments. MP and FB supervised the project. All authors reviewed and approved the final version of the manuscript.

### Conflict of interest statement

The authors declare that the research was conducted in the absence of any commercial or financial relationships that could be construed as a potential conflict of interest. The reviewer YZ and handling Editor declared their shared affiliation.

## References

[B1] Aldred M. J. Crawford P. J. Roberts E. Thomas N. S. (1992). Identification of a nonsense mutation in the amelogenin gene (AMELX) in a family with X-linked amelogenesis imperfecta (AIH1). Hum. Genet. 90, 413–416. 1483698 10.1007/BF00220469

[B2] Bartlett J. D. (2013). Dental enamel development: proteinases and their enamel matrix substrates. ISRN Dent. 2013:684607. 10.1155/2013/684607 24159389 PMC3789414

[B3] Bartlett J. D. Ball R. L. Kawai T. Tye C. E. Tsuchiya M. Simmer J. P. (2006). Origin, splicing, and expression of rodent amelogenin exon 8. J. Dent. Res. 85, 894–899. 10.1177/154405910608501004 16998127 PMC2229627

[B4] Bronckers A. L. Lyaruu D. M. Jansen I. D. Medina J. F. Kellokumpu S. Hoeben K. A.. (2009). Localization and function of the anion exchanger Ae2 in developing teeth and orofacial bone in rodents. J. Exp. Zool. B Mol. Dev. Evol. 312B, 375–387. 10.1002/jez.b.21267 19206174 PMC3142622

[B5] Buchko G. W. Lin G. Tarasevich B. J. Shaw W. J. (2013). A solution NMR investigation into the impaired self-assembly properties of two murine amelogenins containing the point mutations T21–>I or P41–>T. Arch. Biochem. Biophys. 537, 217–224. 10.1016/j.abb.2013.07.015 23896516 PMC3788651

[B6] Chen E. Yuan Z. A. Wright J. T. Hong S. P. Li Y. Collier P. M.. (2003). The small bovine amelogenin LRAP fails to rescue the amelogenin null phenotype. Calcif. Tissue Int. 73, 487–495. 10.1007/s00223-002-0036-7 12958690

[B7] Cho E. S. Kim K. J. Lee K. E. Lee E. J. Yun C. Y. Lee M. J.. (2014). Alteration of conserved alternative splicing in AMELX causes enamel defects. J. Dent. Res. 93, 980–987. 10.1177/0022034514547272 25117480 PMC4293710

[B8] Collier P. M. Sauk J. J. Rosenbloom S. J. Yuan Z. A. Gibson C. W. (1997). An amelogenin gene defect associated with human X-linked amelogenesis imperfecta. Arch. Oral Biol. 42, 235–242. 9188994 10.1016/s0003-9969(96)00099-4

[B9] Delgado S. Ishiyama M. Sire J. Y. (2007). Validation of amelogenesis imperfecta inferred from amelogenin evolution. J. Dent. Res. 86, 326–330. 10.1177/154405910708600405 17384026

[B10] Gibson C. W. Li Y. Daly B. Suggs C. Yuan Z. A. Fong H.. (2009). The leucine-rich amelogenin peptide alters the amelogenin null enamel phenotype. Cells Tissues Organs 189, 169–174. 10.1159/000151384 18701811 PMC2824192

[B11] Gibson C. W. Li Y. Suggs C. Kuehl M. A. Pugach M. K. Kulkarni A. B.. (2011). Rescue of the murine amelogenin null phenotype with two amelogenin transgenes. Eur. J. Oral Sci. 119(Suppl. 1), 70–74. 10.1111/j.1600-0722.2011.00882.x 22243230 PMC3270886

[B12] Gibson C. W. Yuan Z. A. Hall B. Longenecker G. Chen E. Thyagarajan T.. (2001). Amelogenin-deficient mice display an amelogenesis imperfecta phenotype. J. Biol. Chem. 276, 31871–31875. 10.1074/jbc.M104624200 11406633

[B13] Gibson C. W. Yuan Z. A. Li Y. Daly B. Suggs C. Aragon M. A.. (2007). Transgenic mice that express normal and mutated amelogenins. J. Dent. Res. 86, 331–335. 10.1177/154405910708600406 17384027

[B14] Greene S. R. Yuan Z. A. Wright J. T. Amjad H. Abrams W. R. Buchanan J. A.. (2002). A new frameshift mutation encoding a truncated amelogenin leads to X-linked amelogenesis imperfecta. Arch. Oral Biol. 47, 211–217. 11839357 10.1016/s0003-9969(01)00111-x

[B15] Guo J. Lyaruu D. M. Takano Y. Gibson C. W. Denbesten P. K. Bronckers A. L. (2015). Amelogenins as potential buffers during secretory-stage amelogenesis. J. Dent. Res. 94, 412–420. 10.1177/0022034514564186 25535204 PMC4336156

[B16] Hart P. S. Aldred M. J. Crawford P. J. Wright N. J. Hart T. C. Wright J. T. (2002). Amelogenesis imperfecta phenotype-genotype correlations with two amelogenin gene mutations. Arch. Oral Biol. 47, 261–265. 10.1016/S0003-9969(02)00003-1 11922869

[B17] Hart S. Hart T. Gibson C. Wright J. T. (2000). Mutational analysis of X-linked amelogenesis imperfecta in multiple families. Arch. Oral Biol. 45, 79–86. 10.1016/S0003-9969(99)00106-5 10669095

[B18] Kim J. W. Simmer J. P. Hu Y. Y. Lin B. P. Boyd C. Wright J. T.. (2004). Amelogenin p.M1T and p.W4S mutations underlying hypoplastic X-linked amelogenesis imperfecta. J. Dent. Res. 83, 378–383. 10.1177/154405910408300505 15111628

[B19] Kindelan S. A. Brook A. H. Gangemi L. Lench N. Wong F. S. Fearne J.. (2000). Detection of a novel mutation in X-linked amelogenesis imperfecta. J. Dent. Res. 79, 1978–1982. 10.1177/00220345000790120901 11201048

[B20] Kwak S. Y. Green S. Wiedemann-Bidlack F. B. Beniash E. Yamakoshi Y. Simmer J. P.. (2011). Regulation of calcium phosphate formation by amelogenins under physiological conditions. Eur. J. Oral Sci. 119(Suppl. 1), 103–111. 10.1111/j.1600-0722.2011.00911.x 22243235 PMC3448280

[B21] Kwak S. Y. Wiedemann-Bidlack F. B. Beniash E. Yamakoshi Y. Simmer J. P. Litman A.. (2009). Role of 20-kDa amelogenin (P148) phosphorylation in calcium phosphate formation *in vitro*. J. Biol. Chem. 284, 18972–18979. 10.1074/jbc.M109.020370 19443653 PMC2707234

[B22] Lacruz R. S. Habelitz S. Wright J. T. Paine M. L. (2017). Dental Enamel Formation and Implications for Oral Health and Disease. Physiol. Rev. 97, 939–993. 10.1152/physrev.00030.2016 28468833 PMC6151498

[B23] Lagerström M. Dahl N. Nakahori Y. Nakagome Y. Bäckman B. Landegren U.. (1991). A deletion in the amelogenin gene (AMG) causes X-linked amelogenesis imperfecta (AIH1). Genomics 10, 971–975. 1916828 10.1016/0888-7543(91)90187-j

[B24] Lagerström-Fermer M. Nilsson M. Bäckman B. Salido E. Shapiro L. Pettersson U.. (1995). Amelogenin signal peptide mutation: correlation between mutations in the amelogenin gene (AMGX) and manifestations of X-linked amelogenesis imperfecta. Genomics 26, 159–162. 7782077 10.1016/0888-7543(95)80097-6

[B25] Lench N. J. Brook A. H. Winter G. B. (1994). SSCP detection of a nonsense mutation in exon 5 of the amelogenin gene (AMGX) causing X-linked amelogenesis imperfecta (AIH1). Hum. Mol. Genet. 3, 827–828. 8081371 10.1093/hmg/3.5.827

[B26] Lench N. J. Winter G. B. (1995). Characterisation of molecular defects in X-linked amelogenesis imperfecta (AIH1). Hum. Mutat. 5, 251–259. 10.1002/humu.1380050310 7599636

[B27] Le Norcy E. Kwak S. Y. Allaire M. Fratzl P. Yamakoshi Y. Simmer J. P.. (2011). Effect of phosphorylation on the interaction of calcium with leucine-rich amelogenin peptide. Eur. J. Oral Sci. 119(Suppl. 1), 97–102. 10.1111/j.1600-0722.2011.00900.x 22243234 PMC3448291

[B28] Li Y. Suggs C. Wright J. T. Yuan Z. A. Aragon M. Fong H.. (2008). Partial rescue of the amelogenin null dental enamel phenotype. J. Biol. Chem. 283, 15056–15062. 10.1074/jbc.M707992200 18390542 PMC2397487

[B29] Lyaruu D. M. Bronckers A. L. Mulder L. Mardones P. Medina J. F. Kellokumpu S.. (2008). The anion exchanger Ae2 is required for enamel maturation in mouse teeth. Matrix Biol. 27, 119–127. 10.1016/j.matbio.2007.09.006 18042363 PMC2274829

[B30] Martinez-Avila O. M. Wu S. Cheng Y. Lee R. Khan F. Habelitz S. (2011). Self-assembly of amelogenin proteins at the water-oil interface. Eur. J. Oral Sci. 119(Suppl. 1), 75–82. 10.1111/j.1600-0722.2011.00907.x 22243231 PMC6339812

[B31] Pugach M. K. Li Y. Suggs C. Wright J. T. Aragon M. A. Yuan Z. A.. (2010). The amelogenin C-terminus is required for enamel development. J. Dent. Res. 89, 165–169. 10.1177/0022034509358392 20042744 PMC2989847

[B32] Pugach M. K. Suggs C. Li Y. Wright J. T. Kulkarni A. B. Bartlett J. D.. (2013). M180 amelogenin processed by MMP20 is sufficient for decussating murine enamel. J. Dent. Res. 92, 1118–1122. 10.1177/0022034513506444 24072097 PMC3834654

[B33] Ravassipour D. B. Hart P. S. Hart T. C. Ritter A. V. Yamauchi M. Gibson C.. (2000). Unique enamel phenotype associated with amelogenin gene (AMELX) codon 41 point mutation. J. Dent. Res. 79, 1476–1481. 10.1177/00220345000790070801 11005731

[B34] Ryu O. H. Hu C. C. Simmer J. P. (1998). Biochemical characterization of recombinant mouse amelogenins: protein quantitation, proton absorption, and relative affinity for enamel crystals. Connect Tissue Res. 38, 207–214; discussion 241–206. 11063028 10.3109/03008209809017038

[B35] Sekiguchi H. Tanakamaru H. Minaguchi K. Machida Y. Yakushiji M. (2001). A case of amelogenesis imperfecta of deciduous and all permanent teeth. Bull. Tokyo Dent. Coll. 42, 45–50. 10.2209/tdcpublication.42.45 11484794

[B36] Shin M. Hu Y. Tye C. E. Guan X. Deagle C. C. Antone J. V.. (2014). Matrix metalloproteinase-20 over-expression is detrimental to enamel development: a Mus musculus model. PLoS ONE 9:e86774. 10.1371/journal.pone.0086774 24466234 PMC3900650

[B37] Smith C. E. (1998). Cellular and chemical events during enamel maturation. Crit. Rev. Oral Biol. Med. 9, 128–161. 9603233 10.1177/10454411980090020101

[B38] Smith C. E. Warshawsky H. (1977). Quantitative analysis of cell turnover in the enamel organ of the rat incisor. Evidence for ameloblast death immediately after enamel matrix secretion. Anat. Rec. 187, 63–98. 10.1002/ar.1091870106 835843

[B39] Smith C. E. Chong D. L. Bartlett J. D. Margolis H. C. (2005). Mineral acquisition rates in developing enamel on maxillary and mandibular incisors of rats and mice: implications to extracellular acid loading as apatite crystals mature. J. Bone Miner. Res. 20, 240–249. 10.1359/JBMR.041002 15647818

[B40] Snead M. L. Zhu D. H. Lei Y. Luo W. Bringas P. O. Jr. Sucov H. M.. (2011). A simplified genetic design for mammalian enamel. Biomaterials 32, 3151–3157. 10.1016/j.biomaterials.2011.01.024 21295848 PMC3045652

[B41] Tarasevich B. J. Philo J. S. Maluf N. K. Krueger S. Buchko G. W. Lin G.. (2015). The leucine-rich amelogenin protein (LRAP) is primarily monomeric and unstructured in physiological solution. J. Struct. Biol. 190, 81–91. 10.1016/j.jsb.2014.10.007 25449314 PMC4400868

[B42] Xia Y. Ren A. Pugach M. K. (2016). Truncated amelogenin and LRAP transgenes improve Amelx null mouse enamel. Matrix Biol. 52–54, 198–206. 10.1016/j.matbio.2015.11.005 26607574 PMC4873476

[B43] Zhu D. Paine M. L. Luo W. Bringas P. Jr. Snead M. L. (2006). Altering biomineralization by protein design. J. Biol. Chem. 281, 21173–21182. 10.1074/jbc.M510757200 16707492

